# Prevalence, risk factors and antifungal susceptibility pattern of *Candida* species among pregnant women at Debre Markos Referral Hospital, Northwest Ethiopia

**DOI:** 10.1186/s12884-019-2494-1

**Published:** 2019-12-30

**Authors:** Alem Tsega, Feleke Mekonnen

**Affiliations:** 1 Amhara Public Health Institute, P.O.B 641, Bahir Dar, Ethiopia; 2 Department of Medical Laboratory Sciences, School of Health Sciences, College of Medicine and Health Sciences, Bahir Dar University, P O. box: 79, Bahir Dar, Ethiopia

## Abstract

**Background:**

*Candida* is the commonest opportunistic fungi in human. *Candida* species cause diverse types of diseases. Vaginal candidiasis has been reported as one of the most common type of fungal diseases among pregnant women. However; In Ethiopia, due to scarcity of data, much has not been documented regarding the prevalence of *Candida* among pregnant women.

**Objective:**

This study aimed to determine the prevalence, possible risk factors and antifungal susceptibility profile of *Candida* species among pregnant women attending Debre Markos Referral Hospital, Northwest Ethiopia.

**Method:**

A cross-sectional study was conducted from February to May 2017. A total of 384 pregnant women were included using a systematic random sampling technique. Vaginal specimens were collected, inoculated on Candida HiV eg culture Medium and incubated at 37 °C for 24 h.Colonies were identified using standard microbiological methods and selected for further *Candida* Species identification using Hi Chrome agar and germ tube test. Fungal suspensions were made and adjusted at 0.5% MacFarland standard. Modified Kirby-Bauer disk diffusion technique was used for antifungal susceptibility. Data was entered, cleaned using Ep info version 7.1and transported to Statistical Packages for Social Sciences (SPSS) version 21 for analysis. Descriptive statistics and logistic regression were performed. P. value < 0.05 at 95% confidence interval was considered as statistically significant.

**Result:**

From a total of 384 study participants, 96 (25%) were positive for *Candida* species. The predominant *Candida* species was *Candida albicans* 54(56.25%) followed by *Candida krusei* 21(21.9%), *Candida glabrata* 17(17.7%), *Candida tropicalis* 1(1%) and 3(3.1%) were other *Candida* species. Contraceptive use (AOR: 0.394; 95% CI = 0.20–0.74) and prolonged antibiotic uses (AOR: 0.393; 95% CI = 0.21–0.72) were risk factors. All isolates except *Candida krusei* were 100% susceptible to amphotericin-B. Resistance rate was high against itraconazole and Ketoconazole 55(57.3%).

**Conclusion:**

The prevalence of *Candida* species among symptomatic pregnant women was significantly higher than asymptomatic pregnant women. Age group between 26 and 40 years was significantly associated with *Candida* infection. Amphotericin B was the most sensitive antifungal drug. High rate of multiple drugs resistant *Candida* species was detected. Therefore Symptomatic women should be routinely screened and treated.

## Introduction

*Candida* is a fungal pathogen and the commonest opportunistic fungi in human [1]. The genus *Candida* has over 350 heterogeneous species, but only a few of them have been known to cause an opportunistic human disease [2]. Among the various *Candida* species that cause disease in human includes *Candida albicans, Candida glabrata, Candida tropicalis, Candida krusei, Candida parapsilosis, Candida dubliniensis, Candida guillermondii,* and *Candida kyfe* [3–6].. However, *Candida* species are normal floras in mucosal surfaces of the human gastrointestinal tract, genitourinary tract, and mouth. It causes different types of diseases ranging from superficial infection to life threatening invasive and haematogenic infections [7].

Vaginal candidiasis is the most common type of fungal disease all over the world which affects the genital tract of women [8, 9]. They are the main cause of vaginitis next to bacteria and is characterized by vaginal pruritis, thick white vaginal discharge, itching, inflammation of vulva and dyspareunia [10]. Based on the clinical presentation and antifungal response, vaginal candidiasis can be classified as either uncomplicated or complicated. Uncomplicated vaginal *Candidiasis*, mostly caused by *C.albicans* causes mild to moderate symptoms. Whereas complicated vaginal candidiasis is mostly caused by non*-albicans Candida* species and are common among immune-compromised individuals and pregnant women [10].

The disease is more frequently seen among reproductive age group of women. Women of the childbearing age group face at least one episode of vaginal candidiasis in their lifetime [11]. There are several predisposing factors that may increase the development of vaginal candidiasis; these are being diabetes mellitus patient, HIV infection, using contraceptive, pregnancy and broad-spectrum antibiotics [12]. Numbers of gravida and stage of pregnancy have their own contribution to the development of vaginal candidiasis. A study showed that women in their 3rd trimester had the highest infection occurrence [12, 13]. Similarly, multigravida women have been more commonly affected than primigravidae [13].

Although the disease is a common problem among the reproductive age group of women, the incidence of the disease is higher in pregnant women than non-pregnant women because of a number of normal and expected physiological changes that favor the growth of *Candida* in the genitourinary tract [8]. Moreover, during pregnancy the levels of reproductive hormones such as progesterone and estrogen will be elevated which has suppressive effects on the anti-*Candida* activity of neutrophils and inhibits the activity of vaginal epithelial cells respectively [8]. In addition Estrogen decreases immunoglobulins secretion in the vagina resulting an increased vulnerability of pregnant women to vaginal Candidiasis [13, 14] and estrogen also helps to provide high glycogen content in the vagina which serves as a carbon source for *Candida* species [8].

Vaginal candidiasis has different outcomes; the commonest problems are physical and psychological discomfort and suffering. Sometimes the disease itself might be life-threatening [9, 13]. During pregnancy, the problem is more serious since *Candida* colonization associated with preterm birth, infant mortality [15] and pregnant women can even contaminate their infants up to 65%, this will result in invasive neonatal candidiasis [16].

Recently, the incidence of vaginal candidiasis is markedly increasing due to *Candida* species resistant to the commonly used antifungal agent and recurrent infections (11, 14). Recurrent vaginal candidiasis is a relatively frequent condition and may have serious health consequences, like chronic vulvovaginal pain syndrome [17, 18]. The main reason for the increment of non*-albicans Candida* species such as *C.glabrata, C.krusei,* and *C.parapsilosis* is due to extensive use of azole drugs [19]. Studies revealed that the prevalence of candidiasis is high in low economic class and illiterate women [14]. In Ethiopia there is scarcity of information on candidiasis. Therefore this study was designed to estimate the prevalence, associated risk factors and antifungal drug susceptibility profile among pregnant women in one of the district public hospital in northwest Ethiopia.

## Methods

A hospital-based cross-sectional study was conducted from February to June 2017 at Debre Markos Referral Hospital. The hospital is located in Debre Markos town, which is located 300 km northwest of Addis Ababa, the capital city of Ethiopia. Pregnant women who were attending antenatal care at Debre Markos Referral Hospital gynecology and obstetrics clinic were included using systematic random sampling. Women with gynecologic complication, currently on treatment for antifungal therapy, involuntary to participate in the study, and those who were unable to provide vaginal specimen were excluded from the study. Randomization was done after the inclusion criteria, hence 384 study subjects were randomly selected from 1581 pregnant women. Single population proportion was used to estimate the sample size based on the proportion of 50% as there was no previous study conducted in Ethiopia. A confidence level of 95% with an error margin of 0.05 was used for estimation of the sample size.

### Data and sample collection

Socio-demographic status and other possible risk factors were obtained from the study participant using structured questionnaires after informed consent was collected. A vaginal specimen was collected from each participant using a sterile cotton swab moisten by physiological saline then inserted and rotated gently to pick up the specimen [26].

### Laboratory diagnosis

#### Culture of Candida species

The vaginal swab was initially inoculated on Sabouraud Dextrose Agar (Oxoid, UK), then it was incubated at 37 °C for 24 to 48 h. Then white creamy colonies were identified, A fungal suspension on normal saline was made and inoculated onto selective *Candida* HiVeg medium (Hi-Media Laboratories, India) then it was again incubated at 37^0^c for 24 to 48 h. Brown to black pigmented colonies were detected in positive samples. For further identification, the colonies were sub-cultured onto HiChrome agar (Hi-Media Laboratories, India) by incubating at 37 °C for 24 to 48 h. Finally different *Candida* species were identified based on the reaction between specific enzymes of different *Candida* species and a chromogenic substrate which results in the formation of different colony colors [6].

Moreover, Germ tube production in serum was used for identification of *Candida* species [35]. Hence to differentiate *C.albicans* from non*-albicans*, a colony of yeast was added to a sterile test tube which contains 0.5 ml human serum and incubated at 37 °C for 3 h then a loop full sample was placed on a microscopic slide and covered by cover glass. Finally germ tube formation was observed under a microscope [28].

#### Confirmation of identified Candida species

*Candida albicans* had a green colony on Chrome agar and positive for germ tube formation, *C.krusei* formed a pink and spreading colony, *C.tropicalis* formed a dark blue colony while *C.glabrata* colonies appeared as a cream to a white smooth colony.

#### Antifungal susceptibility test

Antifungal susceptibility testing was performed for all *Candida* isolates using a modified disc diffusion method as per Clinical Laboratory Standard Institute guideline by adding 2% glucose and 0.5 μg/mL Methylene Blue dye into Mueller-Hinton agar. A suspension was prepared using normal saline by adding five different colonies and incubating overnight in Sabouraud dextrose agar. Then the suspension was compared to 0.5 McFarland standards. Cotton swab moistened with the fungal suspension was streaked on modified Mueller-Hinton media. Antifungal discs including amphotericin B 100 μg, clotrimazole10μg, fluconazole25 μg, itraconazole10 μg, and ketoconazole10 μg (Oxoid, UK), were placed on Mueller-Hinton agar using disk dispenser, and the plates were incubated at 37^0^C for 24 h. Finally, the zones of inhibition (Zone diameters) were measured and interpreted according to CLSI guideline (M44-A2).

### Data analysis

Data was entered using EpI7 and exported to SPSS Version- 21 statistical package for analyisis. Descriptive statistics, numbers, frequencies, percentages and tables were used to describe the findings. Moreover, bivariable and multivariable logistic regressions were used to assess the association between dependent and independent variables. *P* value less than 0.2 at 95% CI during bivariable analysis was further computed using multivariable logistic regressions to avoid the effect of confounding. Finally *p*-value < 0.05 at 95% CI, was considered statistically significant.

## Results

### Socio-demographic characteristics of study participants

A total of 384 pregnant women were included in this study. The ages of the study participants ranged from 18 to 40 years and the mean age was 25.79 year. Among the study participants, 291 (75.8%) were from urban area. The majority, 371 (96.6%) were married (Table 1).

**Table 1:** Socio-demographic characteristics of study participants at Debre Markos referral hospital, Northwest Ethiopia, May 2017

Characteristics	Frequency	percent
age		
18–25	194	50.5
26–33	164	42.7
34–40	26	6.8
Residence		
Urban	291	75.8
Rural	93	24.2
Educational level		
Illiterate	75	19.5
Primary	50	13
Secondary	101	26.3
Collage/university	158	41.1
Occupation		
Daily labor	20	5.2
Farmer	28	7.3
Governmental employee	104	27.1
House wife	124	32.3
Merchant	82	21.4
Private employee	13	3.4
Student	13	3.4
Marital status		
Single	8	2.1
Married	371	96.6
Divorced	5	1.3
Total	384	100

### Prevalence of vaginal *Candida* species

Of the total 384, 96 (25%) were positive for *Candida* species. The predominant *Candida* species was *Candida .albicans* 54(56.25%) followed by *C.krusei* 21(21.9%). Non-albicans *Candida* species accounts 42 (43.75%) (Table 2). The highest prevalence of *Candida* species was observed in the age range [30–36] year with 10(38.5%) followed by age range 26–33 year 51(31.1%) while; the lowest prevalence was observed in the age range [18–25] years 35(18.0%).

**Table 2:** Frequency of *Candida* species isolated from pregnant women at Debre Markos referral hospital, Northwest Ethiopia, May 2017

Isolates	Number	Percentage
*Candida albicans*	54	56.3
*Candida glabrata*	17	17.7
*Candida tropicalis*	1	1.0
*Candida krusei*	21	21.9
Other /unidentified	3	3.1

### Risk factors

The possible risk factors of *Candida* colonization were assessed including HIV (immuno-compromization), frequent use of contraceptives, prolonged antibiotic use, number of gravidities and gestational period among others. Out of the total of 384 study participants; 40(10.4%) HIV positive, 267(69.5%) contraceptives users, 60(15.6%) antibiotic users and 205(53.4%) Multigravida pregnant women were participated. Of those factors, frequent use of contraceptives (use of oral or injectable contracteptives on daily, monthly or quarterly basis) (AOR: 0.394; 95% CI = 0.20–0.74) and prolonged antibiotic use (taking antibiotics for more than two weeks) (AOR: 0.393; 95% CI = 0.21–0.72) was significantly associated with vaginal Candidiasis with P.value 0.003 and 0.004 respectively (Table 3).

**Table 3:** Association of variables with *Candida* colonization among pregnant women at Debre Markos Referral hospital, Northwest Ethiopia, May2017

Characteristics	*Candida*			Crude-OR (95.0% CI)	Adjusted-OR (95.0% CI)	
	Positive	Negative	Total	OR (Lower-Upper)	OR (Lower-Upper)	*p*-value
18–25	35(18%)	159(82%)	194(50.5%)	1		
26–33	51(31.1%)	113(68.9%)	164(42.7%)	2.050(1.25–3.35)	1.89(1.02–3.49)	.041
34–40	10(38.5%)	16(61.5%)	26(6.8%)	2.839(1.18–6.78)	2.27(1.01–7.30)	.046
Symptom						
Symptomatic	46(40.5%)	67(59.3%)	113(29.4%)	3.035(1.86–4.92)	2.69(1.60–4.52)	<.001
Asymptomatic	50(18.7%)	221(81.5%)	271(70.6%)	1		
antibiotic use						
No	68(21%)	256(79%)	324(84.4)	1		
Yes	28(46.7%)	32(53.3)	60(15.6%)	.034 (0.17–0.53)	.393(0.21–0.72)	.003
Contraceptive use						
No	15(12.8%)	102(87.2%)	117(30.5%)	1		
Yes	81(30.3%)	186(69.7%)	267(69.5%)	.338 (0.18–61)	.394(0.20–0.74)	.004
Gravidity						
Primigravida	36(20.1%)	143(79.9%)	179(46.6%)	1		
Multigravida	60(29.3%)	145(70.7%)	205(53.4%)	.608(0.37–0.97)	.909(0.49–1.67)	.758
HIV status						
Negative	82(23.8%)	262(76.2%)	344(89.6%)	1		
Positive	14(35%)	26(65%)	40(10.4%)	1.720(.85–3.44)	.629(.54–2.71)	.629
Gestational period						
1st trimester	9(23.1%)	30(76.9%)	39(10.2%)	1		
2nd trimester	32(23.7%)	103(76.3%)	135(35.2%)	1.036(.44–2.40)	.937(.37–2.35)	.890
3rd trimester	55(26.2%)	155(75%)	210(54.7%)	1.183(.52–2.64)	1.040(.43–2.50)	.930

### Antifungal susceptibility patterns of *Candida* species

Antifungal susceptibility, testing was performed on 96 vaginal isolates of *Candida* species. All isolates of *Candida* species except *C.krusei* were 100% sensitive for amphotericin-B. Out of 54 *C.albicans* 4(7.4%), 29(53.7%), 38(70.4%), 29(53.7%) were resistant to clotrimazole, fluconazole, ketoconazole and itraconazole, respectively. All isolates of *C.glabrata* were 100% sensitive to fluconazole, whereas; 2(11.8%), 13(76.5%), 7(41.2%) of the isolates were resistant against clotrimazole, itraconazole, and ketoconazole, respectively. The majority, 20(95.2%) of *C.krusei* were sensitive to clotrimazole. One isolate of *C.tropicalis* was sensitive to clotrimazole and fluconazole but it was resistant to itraconazole (Table 4).

**Table 4:** Antifungal susceptibility pattern of *Candida* species isolated from pregnant women at Debre Markos referral hospital, Northwest Ethiopia, May 2017

Antifungal agent	Antifungal susceptibility pattern	*Candida albicans*	*Candida glabrata*	*Candida tropicals*	*Candida krusei*	Others
		Number (%)	Number (%)	Number (%)	Number (%)	Number (%)
amphotericin B	S	54(100)	17(100)	1(100)	15(71.43)	3(100)
	SDD	0(0)	0(0)	0(0)	0(0)	0(0)
	R	0(0)	0(0)	0(0)	6(28.57)	0(0)
Clotrimazole	S	42(77.8)	11(64.7)	1(00)	20(95.2)	3(100)
	SDD	8(14.8)	4(23.53)	0(0)	0(0)	0(0)
	R	4(7.4)	2(11.76)	0(0)	1(4.7)	0(0)
Fluconazole	S	24(44.44)	17(100)	1(100)	18(85.71)	2(66.67)
	SDD	1(1.85)	0(0)	0(0)	1(4.7)	1(33.33)
	R	29(53.7)	0(0)	0(0)	2(9.5)	0(0)
Itraconazole	S	9(16.67)	1(5.88)	0(0)	1(4.7)	0(0)
	SDD	16(29.62)	3(17.64)	0(0)	11(52.38)	0(0)
	R	29(53.7)	13(76.47)	1(100)	9(42.86)	3(100)
ketokonazole	S	8(14.8)	3(17.64)	0(0)	4(19)	1(33.33)
	SDD	8(14.8)	7(41.17)	1(100)	9(42.86)	0(0%)
	R	38(70.37)	7(41.17)	0(0)	8(38)	2(66.67)
Total		54(56.3%)	17(17.7%)	1(1%)	21(21.9%)	3(3.1)

S- Sensitive SDD –Sensitive Dose Dependant R-Resistance

Of the total five antifungal drugs; resistance proportion was highest for itraconazole and ketoconazole with 55(57.3%) (Table 5). Resistance rate was highest among *C.albicans* constituting 37(70.4%). Among *C.glabrata isolates* 2(11.8%) and 7(76.5%), was reported for clotrimazole and itraconazole respectively. From the 96 isolates, Multi-drug resistant *Candida* was confirmed from 35*Candida albicans*, 5*Candida glabrata*, 4*Candida krusei*, and 2unidentified *Candida* isolates (Table 6).

**Table 5:** Total susceptibility pattern of *Candida* species isolated from pregnant women at Debre Markos referral hospital, Northwest Ethiopia, May 2017

Antifungal drug	Sensitive		Intermediate		Resistance	
	Number	%	Number	%	Number	%
Amphotericin B	90	93.8	–		6	6.3
Clotrimazole	77	80.2	12	12.5	7	7.3
Fluconazole	62	64	3	3.1	31	32.3
Itraconazole	11	11.5	30	31.3	55	57.3
Ketokonazole	16	16.7	25	26	55	57.3

**Table 6:** Multiple resistance patterns of *Candida* isolates among pregnant women at Debre Markos Referral Hospital, Northwest Ethiopia, May, 2017

Isolated *Candida*	Antibiogarm patterns					
	Number	R0	R1	R2	R3	R4
*Candida albicans*	54(56.3%)	13(24.07%)	6(11.11%)	7(12.96%)	25(46.29%)	3(5.55%)
*Candida* *glabrata*	17(17.7%)	6(35.29%)	6(35.29%)	2(11.76%)	3(17.64%)	0(0%)
*Candida* *tropicals*	1(1.0%)	0(0%)	1(0%)	0(0%)	0(0%)	0(0%)
*Candida* *krusei*	21(21.9)	5(23.8%)	12((57.14%)	1(4.76%)	3(14.28%)	0(0%)
Others	3(3.1%)	0(0%)	1(33.33%)	2(66.66%)	0(0%)	0(0%)
Total	96(100)	24(25%)	26(27.08)	12(12.5%)	31(32.29%)	3(3.12%)

R0 = No antifungal resistance R1 = Resistant to one antifungal agent R2 = resistant to two antifungal agent R3 = Resistant to three antifungal agent R4 = Resistant to four antifungal agent

## Discussion

The present study showed that the overall prevalence of *Candida* species among pregnant women 25% was similar with a study done in Nigeria (24%) [26] and Saudi Arabia (26%) [28]. While it is less than other studies which was conducted in Cameroon,55.4% [27] and Kenya 90.38% [36]. This great variation might be due to the difference in the study design that the present study includes pregnant women with signs and symptoms of fungal infection.

In the current study the predominant *Candida* species isolated was *C.albicans;* it accounts 56.3% which is comparable with the study done in Nigeria Minna*,* (50%), Kenya *C.albicans* 60(63.83%) [3, 36]. The highest frequency of *Candida* colonization (38.5%) was observed in the age group of [30–36] years, followed by the age group of 26–33(31.1%). This finding is in contrast to a study done in the southwestern part of Nigeria which showed no isolates recorded from the age group above 30 [28]. The difference may be due to environmental, ethnic, socio-economic and cultural factors.

Moreover, the current study showed that age group of women between 26 and 40 was with high *Candida* colonization which is consistent with a study conducted in India 49.6% [29] and Kenya 56(60%) [36]. The reason for this might be related with women at this age group do secrete high concentration of reproductive hormones which can suppress the immune system and creates favorable condition for *Candida* colonization [9]. The other possible reason is related with use of antibiotics which kills bacteria including normal floras thereby *Candida* will have an opportunity to invade the vaginal wall [21].

The present study revealed that prevalence of *Candida* was higher in women who were using contraceptives (30.3%) than non-users (12.8%),this finding was similar with a report in India with 20.51 and 13.52%, respectively [14]. Moreover, this study showed that the use of antibiotics (46.7%) for prolonged duration was the risk (P.value = 0.003) for vaginal colonization with *Candida* species. This finding is consistent with the study conducted in India [29]. The reason might be antibiotic disturbs (eliminates) the normal flora of the human body which is important for defending pathogenic organism as a result high *Candida* colonization will occur [29].

The current findings showed that there were no significant differences between gestational period and *Candida* colonization. The detection rate of *Candida* in the first, second and third trimester were 23.1, 23.7, 26.2% respectively which agreed with a study done in India which shows there was no significant difference between gestational period [25]. However, our findings are in contrast with another study in Kenya which revealed that 3rd trimester had the highest number of vaginal *Candida* species isolated with (68.09%), followed by 2nd trimester (21.28%) and the least number of species was recorded in 1st trimester with (10.63%) [36]. On the other hand study in Nigeria, the proportion of *Candida* was higher in the second trimester (61%) of pregnancy followed by third (21.4%) and first (16.7%) [3]. this difference may be due to the sample size and the study participants that we included were pregnant women with and without symptoms of vaginal infection.

Multigravidae (61.5%) women had a high rate of *Candida* colonization than primigravidae (38.5%). Similarly, a study in Pakistan revealed that multigravidae women more commonly affected than primigravidae the result was (60%) and (40%) respectively [13]. Moreover study in Nepal [24] complements our finding, the reason is related to the rate of infection increases with the number of pregnancy (3rd > 2nd > 1st) thereby frequency of pregnancy reduces immunity; hence *Candida* colonization might be intense.

*Candida* species have different susceptibility to different antifungal agents. In our study, a high resistant rate was observed for itraconazole and ketoconazole with 57.3%. This finding was similar with a study done in Brazil [37].. Moreover; 64% of the isolates were sensitive to fluconazole which agreed with the study done in the Northern part of India [21]. Of the five antifungal drugs which were tested for susceptibility, amphotericin-B was the most effective drug. Our finding was similar with studies in India with 90% [38] and Ghana with 87.2% [39]. The reason for this higher sensitivity of amphotericin-B compared to others is that it is not regularly prescribed and used extensively because of its expensive cost, difficulty to administer it and high toxicity it imposes on kidney. Therefore, the less the drugs are used the less the drug becomes resistant his it is.

The present study showed that clotrimazole was the most effective antifungal drug when compared to other azole group drugs such as ketoconazole, itraconazole, and fluconazole that had 80.2% effectiveness. This finding was complemented with other study conducted in Pakistan which revealed clotrimazole with 70% sensitivity was found to be the most effective antifungal drug [33].

## Conclusions

A high prevalence (25%) of *Candida* species in this study was reported. The predominant species was *C.albicans*, followed by *C.krusei, C.glabrata, C.tropicalis* and other unclassified *Candida* species. With the prevalence of 56.25, 21.9, 17.7, 1 and 3.1% respectively. The proportion of *Candida* from pregnant women with symptoms of vaginal infection was significantly higher than those without signs and symptoms. Moreover, *Candida* prevalence was reported high among age groups in the range of 26–40 years of age. Use of antibiotic for longer duration and contraceptive use were the risk for *Candida* colonization. Antifungal drug susceptibility showed that amphotericin B was the most effective antifungal drug. Most of *Candida* species were resistant to itraconazole and ketoconazole. A high rate of multiple drugs resistant *Candida* species was detected. Therefore, pregnant women should be regularly screened for Candida colonization, proper prophylaxis and treatment should be provided.

## Data Availability

Data that support the findings of this study are also available from the corresponding author upon reasonable request.

## Abbreviations

CLSI: Clinical and Laboratory Standard Institute
GMB: Glucose and Methylene Blue Dye
HIV: Human Immunodeficiency Virus
MHA: Muller Hinton Agar
SPSS: Statistical Package for the Social Sciences

## References

[1] Kumar A, Thakur VC, Thakur S, Kumar A, Patil S (2011). Phenotypic characterization and in vitro examination of potential virulence factors of *Candida* species isolated from a bloodstream infection. W J Sci Techno.

[2] Williams DW, Koriyama T, Silva S, Malic S, Lewis MAO (2011). *Candida* biofilms and oral candidosis: treatment and prevention. Periodontology2000.

[3] Oyewole OA, Okoliegbe IN, Alkhalil S, Isah P (2013). Prevalence of vaginal candidiasis among pregnant women attending the Federal University of Technology, Minna, Nigeria, Bosso clinic. RJPBCS..

[4] Deorukhkar SC, Saini S (2013). Vulvovaginal candidiasis due to non albicans Candida: its species distribution and antifungal susceptibility profile. Int J Curr Microbiol App Sci.

[5] Kumar A, Sharma PC, Kumar A, Negi V (2014). A study on phenotypic traits of *Candida* species isolated from bloodstream infections and in vitro susceptibility to fluconazole. Al Ameen J Med Sci.

[6] Babic M, Hukic M (2010). *Candida albicans* and nonalbicans species as the etiological agent of vaginitis in pregnant and non-pregnant women. BJBMS..

[7] Coutinho HDM (2009). Factors influencing the virulence of *Candida* spp. West Indian Med J.

[8] Kamath P, Pais M, Nayak MG (2013). Risk of vaginal candidiasis among pregnant women. Int J Current Microbiol App Sci.

[9] Esmaeilzadeh S, Omran S, Rahmani M (2009). Frequency and etiology of vulvovaginal candidiasis in women referred to a gynecological Center in Babol, Iran. Int J of Fertility and Sterility.

[10] Hainer BL, Gibson MV (2011). Vaginitis: diagnosis and treatment. American Fam Physi.

[11] Pakshir k YM, Kimiaghalam R (2007). Etiology of vaginal candidiasis in shiraz, southern Iran. Res J Microbiol.

[12] Salehi z SZ, The MAZ (2012). Sensitivity of vaginal isolates of *Candida* to eight antifungal drugs isolated from Ahvaz, Iran. Jundishapur J Microbiol..

[13] Aslam M, Hafeez R, Ijaz S, Tahir M (2008). Vulvovaginal candidiasis in pregnancy. Biomedica..

[14] Aring BJ, Mankodi PJ, Jasani JH (2012). Incidence of vaginal candidiasis in leucorrhoea in women attending in OPD of gynecological and obstetrician department. IJBAR..

[15] Christine LR, Kristen R, George K, Jonathan MM (2011). Treatment of asymptomatic vaginal candidiasis in pregnancy to prevent preterm birth. BMC pre and childbirth.

[16] Bliss JM, Basavegowda KP, Watson AU, Ryan RM (2008). Vertical and horizontal transmission of *Candida albicans* in very low birth weight infants using DNA fingerprinting techniques. Pediatr Infect J.

[17] Schellack N (2012). Recurrent vulvovaginal candidiasis. S Afr Pharm J.

[18] Janković S, Bojović D, Vukadinović D, Daglar E, Janković M, Laudanović D, Lukić V, Mišković V, Potpara Z, Projović I, Čokanović V, Petrović N, Folić M, Savić V (2010). Risk factors for recurrent vulvovaginal candidiasis. Vojnosanit Pregl.

[19] Bokor-Bratiã MB. Oral candidiasis-adhesion of non-albicans candida species. Proc Nat Sci. 2008;(114, 12):69–78.

[20] (Monjaraz–Rodríguez S, Alvarez–Gutiérrez P, V ega–Villa V.M, Xoconostle–Cázares B, Pérez–Luna Y. Prevalence of *Candida* s pp. in women in t he City of Tuxtla Gutierrez , Chiapas . Int Biotech Color J.2012; 2(2): 6–14.

[21] Mohanty S, Xess I, Hasan F, Kapil A, Mittal S, Tolosa E, Prevalence J (2007). Susceptibility to fluconazole of *Candida* species causing vulvovaginitis. Indian J Med Res.

[22] Jombo GTA, Opajobi SO, Egah DZ, Banwat EB, Denen Ak (2010). Symptomatic vulvovaginal candidiasis and genital colonization by Candida species in Nigeria. J Public Health Epidemiol.

[23] Lennox A, Abbey SD, Adiba D, Mboto CI, Ikpoh IS, Akubuenyi FC (2013). Prevalence of vaginitis and vaginosis among the University of Calabar female students. J Public Health Epidemiol..

[24] Shrestha S, Tuladhar NR, Basnyat S, Acharya GP, Shrestha P, Kumar P (2011). Prevalence of vaginitis among pregnant women attending Paropakar maternity and Women's hospital, Thapathali, Kathmandu, Nepal. Nepal Med Coll J.

[25] Parveen N, Munir A, Ikram-Din A, Majeed R (2008). Frequency of vaginal candidiasis in pregnant women attending routine antenatal clinic. J College Phys Surgeons Pakistan.

[26] Al-Nakheel RA, El-Kersh TA, Al-Sheikh YA, Al-Ahmadey ZZ (2013). Prevalence and comparison for detection methods of *Candida* species in vaginal specimens from pregnant and non-pregnant Saudi women. Afr J Microbiol Res.

[27] Toua V, Djaouda M, Gaké B, Menye DE, Christie EA, Tambe E, Akindoh VV, Njiné T (2013). Prevalence of vulvovaginal candidiasis amongst pregnant women in Maroua (Cameroon) and the sensitivity of *Candida albicans* to extracts of six locally used antifungal plants. Int Res J Microbiol.

[28] Donbraye Emmanuel OOB, Dombraye E, Okonkwo IO, Alli JA, Ojezele MO, Nwanze JC (2010). Detection and prevalence of *Candida* among pregnant women in Ibadan Nigeria world. Appl Sci J.

[29] Babin D, Kotigadde S, Sunil Rao P, Rao TV (2013). Clinico-mycological profile of vaginal candidiasis in a tertiary care hospital in Kerala. Inter J Res Biol Sci.

[30] Okonkwo NJ (2010). Prevalence of vaginal candidiasis among pregnant women in Nnewi town of Anambra state, Nigeria. Afr Res Revi.

[31] El-Sayed HM, Hamouda AA (2007). *Candida albicans* causing vulvovaginitis and their clinical response to antifungal therapy. Egyptian J Med Microbiol.

[32] Sasikala G, Agatha D, Jonagold AB, Thenmozhivalli PR (2013). Characterization of *Candida* and its antifungal susceptibility pattern from patients with vaginal candidiasis in a tertiary care hospital in South India. J Pharm Biomed Sci.

[33] Khan F, Baqai R (2010). In vitro antifungal sensitivity of fluconazole, clotrimazole, and nystatin against vaginal candidiasis in females of childbearing age. J Ayub Med Coll Abbottabad.

[34] Wabe NT, Hussein J, Suleman S, Abdella K (2011). In vitro antifungal susceptibility of *Candida albicans* isolates from oral cavities of patients infected with human immunodeficiency virus in Ethiopia. J Exp Integr Med.

[35] Deorukhkar SC, Saini S, Jadhav PA (2012). Evaluation of different media for germ tube production of Candida albicans and Candida dubliniensis. IJBAR.

[36] Nelson M, Wanjiru W, Margaret MW (2013). Prevalence of vaginal candidiasis and determination of the occurrence of Candida species in pregnant women attending the antenatal Clinic of Thika District Hospital, Kenya. OJMM.

[37] Dota KFD, Freitas AR, Consolaro MEL, Svidzinski TIE (2011). A challenge for clinical laboratories: detection of antifungal resistance in *Candida* species causing vulvovaginal candidiasis. Sci..

[38] Arul Sheeba Malar S, Viswanathan T, Malarvizhi A, Lavanya V, Moorthy K (2012). Isolation, characterization and antifungal susceptibility pattern of *Candida albicans* and non-albicans *Candida* from integrated counseling and testing center (ICTC) patients. African J Microbiol Res.

[39] Abruquah HH (2012). Prevalence and antifungal susceptibility of *Candida* species isolated from women attending a gynecological clinic in Kumasi, Ghana. J Sci Techno.

